# TumFlow: An AI Model for Predicting New Anticancer Molecules

**DOI:** 10.3390/ijms25116186

**Published:** 2024-06-04

**Authors:** Davide Rigoni, Sachithra Yaddehige, Nicoletta Bianchi, Alessandro Sperduti, Stefano Moro, Cristian Taccioli

**Affiliations:** 1 Molecular Modelling Section (MMS), Department of Pharmaceutical and Pharmacological Sciences, University of Padova, Via Francesco Marzolo 5, 35131 Padova, Italy; stefano.moro@unipd.it; 2 Department of Animal Medicine, Production and Health, University of Padova, Viale dell’Università 16, 35020 Legnaro, Italy; sachithrakalhari.yaddehige@studenti.unipd.it (S.Y.); cristian.taccioli@unipd.it (C.T.); 3 Department of Translational Medicine, University of Ferrara, Via Luigi Borsari 46, 44121 Ferrara, Italy; nicoletta.bianchi@unife.it; 4 Department of Mathematics “Tullio Levi-Civita”, University of Padova, Via Trieste 63, 35131 Padova, Italy; alessandro.sperduti@unipd.it

**Keywords:** generative model, anticancer molecules, melanoma, SK-MEL-28

## Abstract

Melanoma is the fifth most common cancer in the United States. Conventional drug discovery methods are inherently time-consuming and costly, which imposes significant limitations. However, the advent of Artificial Intelligence (AI) has opened up new possibilities for simulating and evaluating numerous drug candidates, thereby mitigating the requisite time and resources. In this context, normalizing flow models by employing machine learning techniques to create new molecular structures holds promise for accelerating the discovery of effective anticancer therapies. This manuscript introduces *TumFlow*, a novel AI model designed to generate new molecular entities with potential therapeutic value in cancer treatment. It has been trained on the NCI-60 dataset, encompassing thousands of molecules tested across 60 tumour cell lines, with an emphasis on the melanoma SK-MEL-28 cell line. The model successfully generated new molecules with predicted improved efficacy in inhibiting tumour growth while being synthetically feasible. This represents a significant advancement over conventional generative models, which often produce molecules that are challenging or impossible to synthesize. Furthermore, *TumFlow* has also been utilized to optimize molecules known for their efficacy in clinical melanoma treatments. This led to the creation of novel molecules with a predicted enhanced likelihood of effectiveness against melanoma, currently undocumented on PubChem.

## 1. Introduction

Melanoma, a serious form of skin cancer, originates from melanocytes. Melanocytes are cells responsible for producing melanin that colours the skin. It stands as the most severe type of skin cancer due to its potential to metastasize to other body parts if not detected and treated promptly. Individuals with fair skin, blue eyes, and light-coloured hair are predominantly at higher risk, largely due to their lower levels of melanin, making their skin more susceptible to harmful ultraviolet (UV) radiation from the sun [1,2,3,4,5]. Moreover, melanoma poses an increased threat due to its resistance to conventional chemotherapy [6]. Current treatment strategies for melanoma include surgical excision, targeted therapy, and immunotherapy. Targeted therapies are employed for melanomas with specific genetic mutations, such as the BRAF V600E mutation, using inhibitors like vemurafenib and dabrafenib [7]. Immunotherapy, leveraging agents such as anti-PD-1 antibodies (nivolumab and pembrolizumab) and anti-CTLA-4 antibodies (ipilimumab), has shown efficacy in enhancing the immune response against melanoma cancer cells [8,9,10]. The drug discovery and design processes are complex and resource-intensive, often extending over 10–20 years with costs exceeding USD 2 billion [11,12]. Figure S1 presents the number of FDA-approved drugs per year [13], highlighting the small increase in approvals despite investments in research and development increasing each year [14], as can be seen in Figure S2.

In this context, Artificial Intelligence (AI) provides a promising avenue for revolutionizing the field, potentially reducing costs and increasing efficiency. It has become a pivotal tool in various aspects of cancer management, encompassing early detection, precision medicine, imaging, and drug repurposing [15]. Despite these advancements, the complete potential of AI in synthesizing novel anticancer molecules is yet to be fully harnessed and explored [16]. Within AI, machine learning and deep learning are key subfields, including techniques like supervised and unsupervised learning. Supervised learning is utilized for tasks like disease detection and drug efficiency estimation, while unsupervised learning aids in patient stratification and disease recognition [17]. Deep learning, particularly effective in processing large datasets such as those related to the use of images, has contributed notably to melanoma cancer diagnostics among other areas [18,19,20].

Among the unsupervised models, there is a family of approaches that fall under the name of generative models, which are a class of algorithms designed to learn and generate new data that are similar to those within a given training dataset. These models aim to capture the underlying patterns and structures in the training data, enabling them to generate novel samples that share characteristics with the original data. In the field of new drug generation, various approaches based on Variational Autoencoders (VAEs) [21,22,23,24,25,26,27,28,29,30,31], Generative Adversarial Networks (GANs) [32,33,34], Normalizing Flows [11,35,36,37,38,39], and Diffusion Models [40,41,42,43,44,45,46,47,48,49,50] have been explored. Moreover, the advent of large language models (LLMs) using transformer architectures [51] has further expanded this field. Transformer-based models, originally developed for natural language processing tasks, have been successful in capturing complex patterns in data and have been applied in drug generation [52,53,54,55]. Generative models can also include a predictive model to predict the antitumoral activity of generated molecules, enabling the identification of the most promising candidates.

Normalizing flow methods have been applied in various fields, including density estimation [56] and data augmentation [57]. They have been used in tasks such as image generation, speech synthesis, and molecular generation in chemistry. Specific architectures like Real Non-Volume-Preserving (RealNVP) [58] and Glow [59] methods are examples of these. The normalizing flow method represents an effective technique to learn the unknown probability distribution that has generated the data in the training set, i.e., the chemical structure of the molecules. It does this by employing a series of invertible transformations to transmute a probability distribution over input data (i.e., molecule structures) into a designated target probability distribution.

This research incorporates deep learning into drug discovery with *TumFlow*, a novel approach for generating molecular graphs for cancer therapeutics. *TumFlow*, building on the foundational work of MoFlow [11], a pioneering model in the field of models applied to graph structures, adapts and enhances these capabilities specifically to address melanoma treatment challenges. It leverages MoFlow efficient bond and atom generation to create novel molecules aimed to be effective against melanoma cancer cells. When learning to generate new antitumoral molecules, *TumFlow* is trying to solve a complex assignment made of challenging subtasks. The successful generation of useful molecules requires an implicit comprehension of their pharmacokinetics, the identification of single or multiple targets, and the assurance that they bind with high affinity to these to inhibit tumour progression. Each of these subtasks presents formidable difficulties independently, and the fact that the neural network does not have this kind of information to learn from makes the learning process even more challenging.

The integration of *TumFlow* into the drug discovery process reflects a broader trend in AI increasing impact on healthcare and pharmaceutical research. By focusing specifically on melanoma, *TumFlow* addresses a critical need in cancer treatment, offering the potential to rapidly identify and develop new therapeutic molecules. For this reason, this work represents a novel contribution to anticancer drug discovery.

## 2. Results and Discussion

In the following, some novel molecules generated by *TumFlow* against the SK-MEL-28 melanoma tumour are presented, while its limitations are discussed in Section S7 of the Supplementary Material. Two processes were adopted for generating new molecules with *TumFlow* that will be individually discussed in the following. Each molecule will be introduced with its predicted GI50 score, the normalized SAS [60] value, and its similarity measure in relation to the initial molecule. The similarity score allows evaluation of how much the newly generated molecule and the starting molecule differ from each other. This score is calculated using the Tanimoto similarity of the Morgan Fingerprint [61]. More details about the generation process adopted by *TumFlow* and the metrics considered in this work are reported in Section 3.

### 2.1. Generation Starting from the NCI-60 Dataset

This section presents a chosen set of novel molecules generated by *TumFlow* considering molecules from the training set as starting points. Specifically, in this generation procedure, the first 140 molecules with higher antitumoral efficacy appearing in the training set, i.e., antitumoral molecules tested in vitro from the NCI-60 project [62], were used as a starting point.

**Figure 1:**
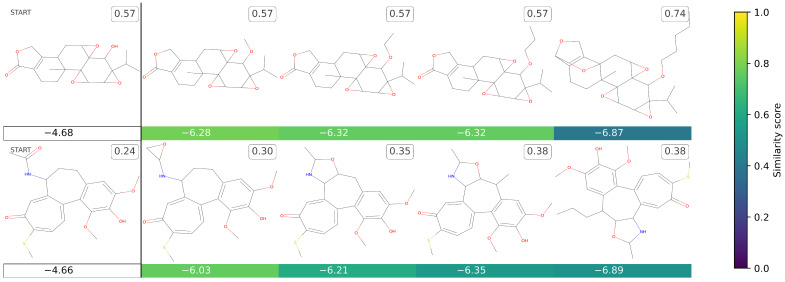
This grid presents the novel molecules that *TumFlow* generated starting from those in the dataset. The first column on the left reports the starting molecule structures, while the other columns report the new molecules. The score reported under each generated molecule represents the *TumFlow* predicted GI50 score, while the colour conveys the similarity score of the newly generated structure in relation to the starting molecule structure. The number to the top right of each molecule is the normalized SAS score.

Figure 1 presents some molecules obtained from a provided starting molecule, while the corresponding canonical SMILES [63,64,65] are reported in Table S2. Specifically, the figure presents a grid where the first column on the left depicts the starting molecule structures, while the other columns report the new molecules obtained from them. By inspecting the molecular structures and the corresponding predicted GI50 scores, it can be seen that *TumFlow* attempts to enhance the structure of the provided starting molecule to improve its efficacy against melanoma tumours. Nevertheless, in the process, the model tends to generate increasingly complex molecules, introducing synthesis issues, and molecular structures dissimilar from the starting one.

**Figure 2:**
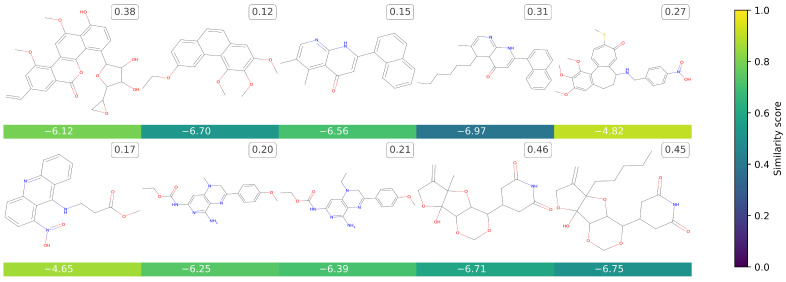
This grid presents the novel molecules that *TumFlow* generated starting from those in the dataset. The score reported under each generated molecule represents the *TumFlow* predicted GI50 score, while the colour conveys the similarity score of the newly generated structure in relation to the starting molecule structure. The number to the top right of each molecule is the normalized SAS score.

Contrary to Figure 1, Figure 2 presents a few molecules obtained by the whole generation process, i.e., considering many starting structures. The corresponding canonical SMILES, including those of the provided starting molecules, are reported in Table S3. All of these molecules are chemically noteworthy and interesting, particularly due to their absence in the dataset. It is important to note that the generation process sometimes results in uncommon molecules that encounter challenges in synthesis and/or contain rare substructures. Nevertheless, thanks to the SAS score, it becomes possible to identify molecules that are challenging to synthesize and, consequently, filter them as necessary.

None of the novel molecules, except the first compound (CID = 121297650) generated by the first molecule reported in Figure 1, are available in PubChem [66]. This absence of novel molecules in PubChem highlights the pioneering nature of *TumFlow* in exploring unknown chemical spaces, while the presence of the already-existing molecule demonstrates the ability of the model to generate meaningful structures.

### 2.2. Generation Starting from Clinically Adopted Anti-Melanoma Molecules

Herein, a selected set of novel molecules generated by *TumFlow* considering clinical molecules as starting points are presented. Specifically, some of the molecules reported in Table S1, known for their efficacy in clinical treatments for melanoma, were used as starting points.

**Figure 3:**
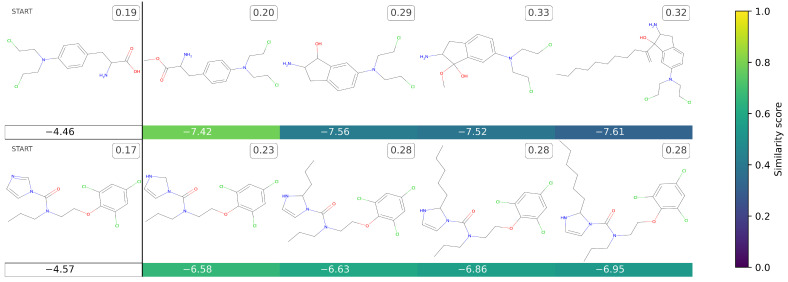
This grid presents the novel molecules that *TumFlow* generated starting from two clinical molecules. The first column reports the starting molecule structures, while the other columns report the new molecules. The score reported under each generated molecule represents the *TumFlow* predicted GI50 score, while the colour conveys the similarity score of the newly generated structure in relation to the starting molecule structure. The number to the top right of each molecule is the normalized SAS score.

Figure 3 presents some novel molecules, while the corresponding canonical SMILES are reported in Table S4. Regarding the molecules generated from clinical drugs, a pattern akin to those originating from in vitro molecules is discernible. In this scenario as well, *TumFlow* demonstrates the capacity to generate novel molecular structures, albeit occasionally encountering challenges in synthesis. Notably, with the exception of just one molecule, all the newly generated structures are absent from PubChem. In fact, the initial molecule derived from the first clinical drug, specifically the second structure in the first row of the image, has been identified as a previously studied compound against cancer, bearing the corresponding NSC = 133726 and CID = 421441. More precisely, this compound has already been subjected to several **in vivo testing** on mice, demonstrating its activity against the leukemia cell line L1210 (e.g., PubChem AID = 248).

The identification of a novel molecule, previously studied for its anticancer properties and **not present in the training set**, underscores *TumFlow*’s potential ability to explore the chemical space beyond the confines of existing datasets. This capability suggests that *TumFlow* has the capacity to propose compounds with therapeutic relevance that might not have been part of the original training data, stressing its potential to contribute to the discovery of compounds with valuable properties.

### 2.3. Benchmarking

During the development and evaluation of *TumFlow*, we encountered significant challenges: the absence of a benchmarking dataset suitable for model validation and the intrinsic problem of synthesizing new molecules. Benchmark datasets play a crucial role in the field of machine learning, providing a base against which newly developed models can be tested and compared. These datasets enable researchers to assess the accuracy, efficiency, and overall performance of their models in a standardized context. Unfortunately, in the context of generating novel molecular entities for treating the SK-MEL-28 tumour, such a benchmarking dataset does not exist.

The lack of a benchmark could be compensated by experimental validation. Yet, experimentally validating the molecules produced by *TumFlow* also presents its challenges. As *TumFlow* designs new molecular structures with potential anticancer properties, most of the candidate molecules lack prior synthesis or testing documentation. Consequently, before any biological efficacy testing can start (e.g., assays on melanoma cell lines), these molecules must first be synthesized. Synthesizing new molecules is not only a complex process that demands specialized expertise but also involves substantial time investment and significant financial costs.

These challenges present a substantial hurdle for validating the effectiveness of generative approaches in generating therapeutically valuable molecules.

### 2.4. Code Implementation

The implementation of the *TumFlow* model, along with the code utilized for training and generating novel molecules and a comprehensive user configuration guide, are openly available at https://github.com/drigoni/TumFlow (accessed on 6 February 2024). Within the repository, there are all the necessary scripts for result reproducibility, enabling robust verification of findings. Furthermore, the repository hosts the trained weights of the *TumFlow* model as well as the dataset, including all GI50, IC50, LC50 and TGI scores used in this work. Additionally, a Docker [67] container is provided to streamline usage across various computing environments, ensuring accessibility and ease of deployment.

## 3. Materials and Methods

*TumFlow* is based on MoFlow, a normalizing flow model originally developed for the generation of graph structures without any focus on anticancer molecules. On the other hand, *TumFlow* aims to learn the unknown probability distribution that has generated the chemical structures of the molecules in the dataset, with the purpose of using the learned distribution to generate new novel chemical structures that should convey similar substructures and similar anticancer activities. Therefore, *TumFlow* is developed to predict new antitumour molecules against the SK-MEL-28 melanoma, addressing all the unique challenges and requirements of melanoma treatment. It is trained on the comprehensive NCI-60 dataset, made public by the National Cancer Institute [68], which encompasses thousands of molecules tested across a broad spectrum of tumour cell lines.

The following sections will present in more detail the data preprocessing method applied to the NCI-60 dataset, as well as the *TumFlow* model. Additional details about normalizing flows are reported in Section S3 of the Supplementary Material, while more details on the *TumFlow* model are reported in Section S4.

The following mathematical notations are adopted:

(i) Lower-case symbols for scalars, indexes, and assignment to random variables, e.g., *n* and *x*;
(ii) Italic upper-case symbols for sets and single random variables, e.g., *A* and *X*;
(iii) Bold lower-case symbols for vectors and assignments to vectors of random variables, e.g., a and x;
(iv) Bold upper-case symbols for matrices, tensors, and vectors of random variables, e.g., A and Z;
(v) The position within a tensor or vector is denoted by numeric subscripts in square brackets, for example, $A_{\left[i,a:b,:\right]}$, where $i,a,b\in N^{+}$, and “:” indicates the positions from *a* to *b*. The solitary use of the colon symbol “:” represents all positions;
(vi) Calligraphic symbols for domains, e.g., Q;
(vii) When it is clear from the context, the probability random variables are omitted, as $P\left(x\right)$ instead of $P\left(X=x\right)$.

### 3.1. Data Sources and Data Preprocessing

The NCI-60 project [62], launched in 1990, employs 60 human tumour cell lines representing diverse cancers to evaluate up to 7000 small molecules annually for anticancer properties. It provides four files with the results of their experiments: “GI50.csv”, “LC50.csv”, “IC50.csv”, and “TGI.csv”. In this work, only the “GI50.csv” file was used. It contains data on the GI50 (Growth Inhibition of 50%) values, which are derived from laboratory assays that measured the concentration of a chemical compound required to inhibit the growth of a specific tumour cell line by 50%. Specifically, the GI50 values are obtained by interpolating the *GIPRCNT* scores, which are the percentage of treated cell growth as a fraction of control cell growth, corrected for the count of cells at the time of drug addition in the assay. A score of 100 is control growth, 0 is complete inhibition of growth (cytostasis), and −100 is complete cell kill. Thus, these values serve as a direct indicator of the compound’s potential antitumoral efficacy as a lower GI50 value indicates a higher efficacy of the molecule in inhibiting tumour growth in the tested cells. More information is reported in the NCI-60 project website. In addition, the dataset includes the National Service Center (NSC) code, a unique numeric identifier assigned to substances tested and evaluated by the National Cancer Institute, and information on the tested cell line.

The choice of this file was based on its relevance in identifying compounds with potential antitumoral efficacy, particularly in the context of SK-MEL-28 melanoma cells. In fact, a preliminary data analysis visible in Figure S3 revealed that molecules used clinically show better representation in the GI50 dataset. Indeed, the GI50 scores offer a more accurate representation of clinical drugs since they exhibit a more evenly distributed pattern and better distinguish the effects of various drugs. Additionally, the violin plot illustrating the mass of GI50 scores demonstrates greater variability than that of the IC50 scores, which, conversely, appear more condensed. However, even though the presented work focuses on the GI50 score, other indicators like the IC50 can also be utilized seamlessly. This correlation reinforces the validity of the approach presented in this work and highlights the importance of integrating real and clinically relevant data into the modelling process.

The training of the *TumFlow* model was performed on this data, focusing only on molecules tested on SK-MEL-28 melanoma cell lines made by chemical elements commonly found in organic compounds (only molecules composed by hydrogen (H), carbon (C), nitrogen (N), oxygen (O), fluorine (F), phosphorus (P), sulphur (S), chlorine (Cl), selenium (Se), bromine (Br), and iodine (I)). During the data preprocessing phase, all molecules with a positively charged oxygen (O^+^) were removed, and all “ion pair” compounds were sanitized, selecting only the largest connected component as the main molecule structure while discarding the remaining smaller component(s). If the sanitization resulted in a structure already existing in the dataset, only the experiment with the highest efficacy score was retained. For molecules with multiple in vitro experiments, the corresponding GI50 values were averaged. Following the data preprocessing phase, the dataset consists of 46,766 unique molecules, each paired with its corresponding GI50 efficacy value.

### 3.2. TumFlow

*TumFlow* aims to predict new anticancer molecules by exploiting the graph representation of the molecule structure, differently from other works adopting the SMILES sequential representation of the molecule, such as [22,23].

Mathematically, let D be the dataset, Tr be the training set of molecules, $\Theta_{n}$ and $\Theta_{e}$ be, respectively, the set of atom types and the set of edge types extracted from dataset D. Let $d_{v}=|\Theta_{n}|$ be the number of atom types, $d_{e}=|\Theta_{e}|$ be the number of edge types, and $d_{n}$ the maximum number of atoms, hydrogens excluded, forming the molecules in dataset D. Then, a molecule is represented as a graph G∈G:

$$\begin{array}{cc} G & =(V,E), \end{array}$$

where $V\in {\left\{0,1\right\}}^{d_{n}\times d_{v}}$ is a node-type matrix and $E\in {\left\{0,1\right\}}^{d_{n}\times d_{n}\times d_{e}}$ is an edge-type tensor, such that $V_{i,v}=1$ only if the molecule node *i* is of type *v* and such that $E_{i,j,e}=1$ only if the molecule nodes *i* and *j* are connected through a bond of type *e*. The set of all possible graphs is defined as follows:

$$\begin{array}{cc} G & =V\times E={\left\{0,1\right\}}^{d_{n}\times d_{v}}\times {\left\{0,1\right\}}^{d_{n}\times d_{n}\times d_{e}}. \end{array}$$

**Figure 4:**
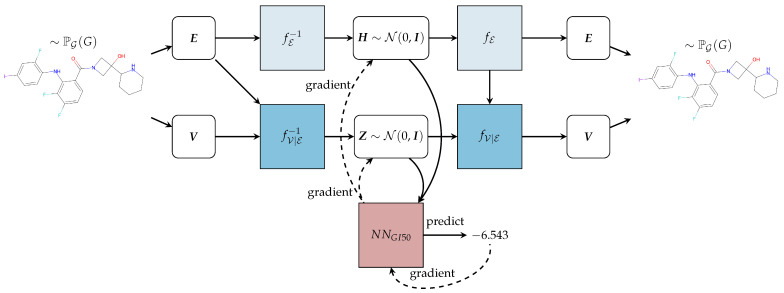
Overview of the *TumFlow* model. From the molecule in input (on the left) the graph G=(V,E) is constructed, and both the latent representations $H=f_{E}^{-1}\left(E\right)$ and $Z=f_{V|E}^{-1}\left(V;E\right)$ are obtained. From the latent representations, the graph *G* and then the molecule in output (on the right) are reconstructed through the functions $f_{E}$ and $f_{V|E}$. The module $NN_{GI50}$ predicts the GI50 score and can optimize the molecule structure, employing the gradient descent approach.

*TumFlow* aims to learn the complex probability distribution $P_{G}$, from which the molecules in the dataset are generated, in order to sample from it new useful molecule graphs $G∼P_{G}\left(\tilde{G}\right)$. $\tilde{G}$ denotes the random variable over graph structures with support in G. *TumFlow* factorizes the probability distribution as follows:

$$\begin{array}{c} P_{G}\left(\tilde{G}\right)=P_{G}\left((\tilde{V},\tilde{E})\right)=P_{V}\left(\tilde{V}|\tilde{E}\right)\cdot P_{E}\left(\tilde{E}\right), \end{array}$$

where $P_{E}\left(\tilde{E}\right)$ is the probability distribution over molecule bounds, $P_{V}\left(\tilde{V}|\tilde{E}\right)$ is the conditioned probability distribution over atoms given molecule bounds, and both $\tilde{E}$ and $\tilde{V}$ are vectors of random variables. In simpler terms, *TumFlow* first predicts the set of bonds forming the structure of the molecule and then conditions the generation of the molecule atoms by the predicted bonds. It uses two jointly trained normalizing flow models. The first is used to predict the molecule bonds and the second is used to predict the molecule atoms. The decision to factorize the full probability distribution as predicting the bonds forming the molecule’s structure before predicting the atoms is purposeful. This factorization enables the effective utilization of graph neural networks (more on this in the subsequent paragraphs). In graph neural networks, nodes update their states based on information propagated through the edges. Thus, by first predicting the bonds (edges), a foundation is established upon which the subsequent prediction of atoms (nodes) can be informed and influenced. This approach aligns well with the nature of molecular structures, where the connectivity between atoms greatly influences their properties and behaviours. Figure 4 reports the overall model architecture, summarizing the main steps.

*TumFlow* is trained to optimize the negative log-likelihood loss:

$$\begin{array}{cc} L_{G}\left(D\right) & =-\frac{1}{|D|}\sum_{G\in D}\log P_{G}\left(G\right);\\ \; & =-\frac{1}{|D|}\sum_{(V,E)\in D}\log P_{V}\left(V|E\right)+\log P_{E}\left(E\right); \end{array}$$

with

$$\begin{array}{cc} \log P_{E}\left(E\right)\; & =\log P_{H}\left(H\right)-\log\left|\det\left(\frac{\partial f_{E}\left(H\right)}{\partial H}\right)\right|;\\ \log P_{V}\left(V|E\right)\; & =\log P_{Z}\left(Z|E\right)-\log\left|\det\left(\frac{\partial f_{V|E}\left(Z;E\right)}{\partial Z}\right)\right|; \end{array}$$

where $f_{E}$ and $f_{V|E}$ are two invertible and differentiable functions to learn, H and Z are, respectively, two latent representations for atom and adjacency tensors, and $P_{H}$ and $P_{Z}$ are the two simple target distributions, i.e., two standard normal distributions N(0,I), with zero mean and identity matrix as covariance matrix.

Affine coupling layers are used in the implementation of both $f_{V|E}=\Phi_{l_{V|E}}\circ \ldots \circ \Phi_{1}$ and $f_{E}=\Psi_{l_{E}}\circ \ldots \circ \Psi_{1}$, where $l_{V|E}$ and $l_{E}$ represent the number of coupling layers composing $f_{V|E}$ and $f_{E}$, respectively. For the sake of clarity, the explicit dependency on E in the notation of $\Phi_{i}$ is omitted. In the implementation of the coupling layers, the sigmoid function replaces the exponential function, as it provides better numerical stability when stacking multiple coupling layers. Mathematically, each function $\Phi_{i}^{-1}:R^{d_{n}\times d_{v}}\to R^{d_{n}\times d_{v}}\forall i\in \left\{1,\ldots ,l_{V|E}\right\}$ splits the input into two parts according to the node types dimension $d_{v}$. Given $Z^{i-1}=\Phi_{i}^{-1}\left(Z^{i}\right)$ and a selected dimension $\tilde{d_{v}}$:

$$\begin{array}{c} Z^{i-1}=\left\{\begin{array}{cc} Z_{\left[:,1:\tilde{d_{v}}\right]}^{i-1} & =Z_{\left[:,1:\tilde{d_{v}}\right]}^{i}\;;\\ Z_{\left[:,\tilde{d_{v}}+1:d_{n}\right]}^{i-1} & =Z_{\left[:,\tilde{d_{v}}+1:d_{n}\right]}^{i}⊙sig\left(s_{V|E}^{i}\left(Z_{\left[:,1:\tilde{d_{v}}\right]}^{i}\right)\right)+t_{V|E}^{i}\left(Z_{\left[:,1:\tilde{d_{v}}\right]}^{i}\right); \end{array}\right. \end{array}$$

where $Z=Z^{0}$ and $V=Z^{l_{V|E}}$. Functions $s_{V|E}^{i}$ and $t_{V|E}^{i}$ are multi-layer perceptions (MLPs) based on the output of a graph neural network [69], which aims to learn the representation of the graph underlying the molecular structure.

Similarly, each function $\Psi_{i}^{-1}:R^{d_{n}\times d_{n}\times d_{e}}\to R^{d_{n}\times d_{n}\times d_{e}}\forall i\in \left\{1,\ldots ,l_{E}\right\}$ splits the input into two parts according to the bond types dimension $d_{e}$. Given $H^{i-1}=\Psi_{i}^{-1}\left(H^{i}\right)$ and a selected dimension $\tilde{d_{e}}$,

$$\begin{array}{c} H^{i-1}=\left\{\begin{array}{cc} H_{\left[:,:,1:\tilde{d_{e}}\right]}^{i-1} & =H_{\left[:,:,1:\tilde{d_{e}}\right]}^{i}\;;\\ H_{\left[:,:,\tilde{d_{e}}+1:d_{e}\right]}^{i-1} & =H_{\left[:,:,\tilde{d_{e}}+1:d_{e}\right]}^{i}⊙sig\left(s_{E}^{i}\left(H_{\left[:,:,1:\tilde{d_{e}}\right]}^{i}\right)\right)+t_{E}^{i}\left(H_{\left[:,:,1:\tilde{d_{e}}\right]}^{i}\right), \end{array}\right. \end{array}$$

where $H=H^{0}$ and $E=H^{l_{E}}$. Functions $s_{E}^{i}$ and $t_{E}^{i}$ are implemented with a sequence of 2D convolutional neural networks.

It is essential to consider that the normalizing flow framework is designed for continuous space values, and, as such, it cannot be directly applied to discrete structures like node and adjacency tensors. To address this limitation, a pre-processing step is implemented before the utilization of coupling layers. Specifically, a random uniform noise drawn from a carefully selected interval of values is added to each entry of the tensors. This introduction of noise serves the purpose of incorporating a continuous element into the discrete structures, aligning them with the framework requirements and enabling the subsequent application of coupling layers. The carefully selected noise distribution enables the accurate selection of corresponding atoms and bonds through the argmax function when utilizing $f_{V|E}$ and $f_{E}$ to reconstruct *G*.

#### 3.2.1. Prediction of the GI50 Scores

*TumFlow* includes a nonlinear neural network $NN_{GI50}$ designed to predict the antitumour activity of individual molecules. This neural network is learned once the main normalizing flow networks have been learned. More precisely, the neural network is trained to predict the GI50 score associated with the molecule’s latent representation. Mathematically, $NN_{GI50}$ represents the following function:

$$\begin{array}{c} NN_{GI50}:G\to R. \end{array}$$

This function is trained with the mean squared error (squared L2 norm) loss computed among predicted values and values measured in vitro. In more detail, given a molecule graph *G*, its predicted antitumour activity *p* is estimated as follows:

$$\begin{array}{cc} p & =NN_{GI50}\left(f_{vc}\left(H,Z\right)\right);\\ H & =f_{E}^{-1}\left(E\right);\\ Z & =f_{V|E}^{-1}\left(V;E\right); \end{array}$$

where $f_{vc}$ is a function that linearizes both the tensors H and Z, and then concatenates them together.

#### 3.2.2. New Molecule Generation

After the learning phase, *TumFlow* can generate novel chemical structures conveying similar antitumoral activities to those in the training set and, using $NN_{GI50}$, it can also predict the antitumoral activity for each molecule. While this approach used alone proves highly valuable in other research domains, such as computer vision, where realistic face images need to be generated [70], it shows limitations when creating new antitumoral molecules. In fact, unlike scenarios where realistic faces are generated from datasets comprised of numerous real-world face images, the NCI-60 dataset includes many molecules with suboptimal antitumour efficacy. Consequently, employing this simplistic method to develop new molecules may yield structures with limited antitumour activities. *TumFlow* generates new molecules adopting a different approach that closely resembles structure optimization. Starting from a molecule with high antitumoral efficacy, through the use of the $NN_{GI50}$ neural network, it modifies its structure to exhibit better antitumoral effects. Given a molecule in input, the optimization takes place many times, and for each of them *TumFlow* predicts the new molecule structure with associated predicted GI50 scores.

**Figure 5:**
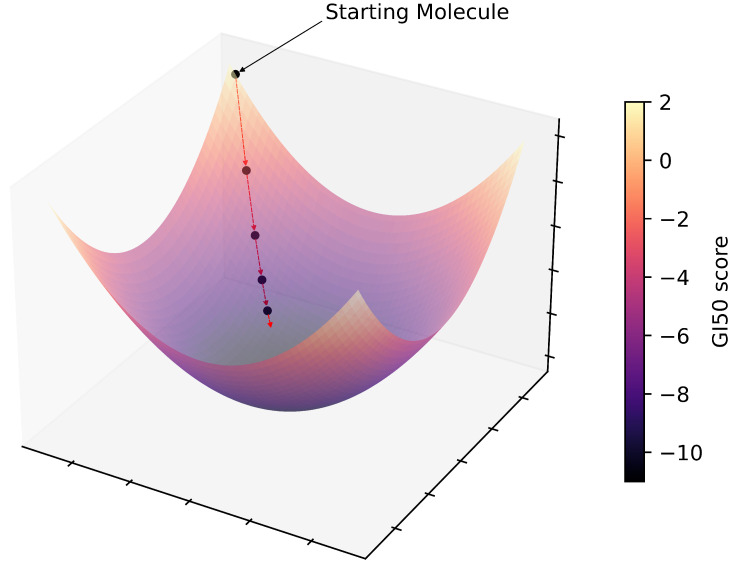
Figure representing the main idea behind the *TumFlow* generation of novel molecules with lower GI50 values, i.e., high antitumoral efficacy. Starting from an initial good molecule structure, new molecules are sampled in compliance with the gradient descent approach.

The optimization, which can also be seen in Figure 5 and Figure S4, is performed using the gradient descent method, which is a pervasive optimization algorithm in machine learning employed to reduce the value of an objective function. Its primary objective is to iteratively approach the minimum of a function by moving in the direction of the most significant decrease in that function. In other words, *TumFlow* applies the gradient descent technique to the function $NN_{GI50}$, aiming to minimize the GI50 score. The optimization starts from a given molecule, i.e., the black ball in the top part of the image. Then, $NN_{GI50}$ predicts the GI50 score associated with the starting molecules, as well as the direction to follow in the latent space to minimize the score. In other words, the direction to follow is the negative of the gradient returned by the $NN_{GI50}$ component, as the lower the value is, the better the antitumoral properties are. Consequently, a new point in the latent space is selected, which can be decoded back to a molecule structure through $f_{E}$ and $f_{V|E}$. This process is repeated several times. *TumFlow* performs the optimization process outlined above for each molecule, taking into account various gradient descent step values. In other words, for each optimization process, the movement performed in latent space involves making jumps of varying distance.

The generation of new molecules is performed following two different approaches:

(i) In the first approach, the starting point consists of molecules with higher antitumoral efficacy appearing in the training set, i.e., antitumoral molecules tested in vitro from the NCI-60 project;
(ii) In the second approach, the starting point consists of nine molecules, reported in Table S1, known for their efficacy in clinical treatments for melanoma.

In the process of generating new molecules, *TumFlow* is equipped with the ability to assess the Synthetic Accessibility Score (SAS) of drug-like molecules based on molecular complexity and fragment contributions [60]. This incorporation is essential because, even though *TumFlow* allows the generation of novel molecules, sometimes it produces energetically unstable structures and complex molecules that are challenging to synthesize. Further details are discussed in Section S7 of the Supplementary Material. In this work, the reported SAS values are normalized to fall within the range of [0,1], with lower values indicating greater ease of molecule synthesis and higher values suggesting increased difficulty in the synthesis process. The incorporation of this metric significantly enhanced the quality of molecules generated by *TumFlow*, facilitating the identification of compounds with potential antitumour effectiveness as well as a desired level of synthesis complexity.

## 4. Conclusions and Future Prospects

This investigation prominently showcases the generative capabilities and potential of *TumFlow* in oncological drug development. Unlike conventional methodologies, the presented approach harnesses the distinctive strengths of normalizing flow algorithms, notably their adeptness at modelling complex molecule distributions and generating accurate new data samples. This marks a substantial leap beyond traditional AI techniques, delivering unprecedented precision and efficiency in generating novel anticancer molecules.

In particular, this work demonstrates that *TumFlow* can identify crucial patterns and correlations between molecular structures and their predicted effectiveness against tumours like melanoma. It not only exhibits creativity in generating novel and promising molecules that have not been seen before but also has the capability to generate molecules not included in the training dataset, which already exist and have been subjected to in vivo experiments for antitumoral assessment. Although this creativity is essential for generating new drugs, there are some limitations that come with it, such as the feasibility of synthesis and the chemical instability of some generated structures.

By redefining the boundaries of possibilities within normalizing flow algorithms, the *TumFlow* model emerges not merely as a predictive instrument but as a designer, hopefully, of future oncological therapies. This methodology promises to diminish the time and financial constraints associated with drug discovery, steering researchers toward an era where swift, targeted, and potent cancer therapies are not merely conceivable but attainable. The *TumFlow* model, code, and documentation on GitHub [71] enhance reproducibility and accessibility in molecular generation. With scripts, trained weights, datasets, and a Docker container provided, it is a valuable resource for drug discovery research.

In summary, the application of the *TumFlow* model, as presented in this study, represents a significant advancement in the fight against cancer. This endeavour not only exemplifies the model’s current achievements but also paves the way for a myriad of advancements in anticancer treatments and patient care.

Future research should consider various strategies to advance and increase the efficacy of the model presented in the context of oncological studies. One of these is the integration of broader and more diverse datasets, encompassing a wide variety of cancer types and molecules. Indeed, by exploiting drugs addressing different types of cancer, the model could learn complementary information that could enhance the discovery of new and more effective molecules. Moreover, given *TumFlow*’s tendency to generate energetically unstable complex structures, future works will consider the inclusion of the SAS values during the generation process and the inclusion of metrics to account for the energetic stability of the molecule. Additionally, incorporating information on the inhibition, lethality, and toxicity of antitumour molecules could provide an even more accurate algorithm for predicting new anticancer drugs. Another critical aspect is the continual enhancement of the computational and algorithmic capabilities of the model, to tackle challenges like interpreting molecular mechanisms and predicting drug side effects and resistance with the purpose of optimizing the molecules to yield better properties.

## Data Availability

Data is contained within the article and the Supplementary Materials. The code is openly available at https://github.com/drigoni/TumFlow (accessed on 6 February 2024).

## Supplementary Materials

References [72,73] are cited in the supplementary materials. The following supporting information can be downloaded at: https://www.mdpi.com/article/10.3390/ijms25116186/s1.
